# A Role for the Ubiquitin Ligase Nedd4 in Membrane Sorting of LAPTM4 Proteins

**DOI:** 10.1371/journal.pone.0027478

**Published:** 2011-11-11

**Authors:** Ruth Milkereit, Daniela Rotin

**Affiliations:** Program in Cell Biology, The Hospital for Sick Children, Biochemistry Department, University of Toronto, Toronto, Ontario, Canada; University of Hong Kong, Hong Kong

## Abstract

**Background:**

The Lysosome associated protein transmembrane (LAPTM) family is comprised of three members: LAPTM5, LAPTM4a and LAPTM4b, with the latter previously shown to be overexpressed in numerous cancers. While we had demonstrated earlier the requirement of the E3 ubiquitin ligase Nedd4 for LAPTM5 sorting to lysosomes, the regulation of sorting of LAPTM4 proteins is less clear.

**Methodology/Principal Findings:**

Here we show that LAPTM4a and LAPTM4b are localized to the lysosome, but unique to LAPTM4b, a fraction of it is present at the plasma membrane and its overexpression induces the formation of actin-based membrane protrusions. We demonstrate that LAPTM4s, like LAPTM5, are able to co-immunoprecipitate with the E3 ubiquitin ligase Nedd4, an interaction that is dependent on LAPTM4 PY motifs and plays a role in membrane sorting. Accordingly, in Nedd4 knockout mouse embryonic fibroblasts (MEFs), LAPTM4a and LAPTM4b show reduced lysosomal localization. Moreover, lack of PY motifs leads to enhanced missorting of LAPTM4b to the plasma membrane instead of the lysosome.

**Conclusions/Significance:**

These results suggest that while some requisites of LAPTM5 lysosomal sorting are conserved among LAPTM4 proteins, LAPTM4a and LAPTM4b have also developed distinct sorting requirements.

## Introduction

The Lysosome Associated Protein Transmembrane (LAPTM) family of proteins include LAPTM4a, LAPTM4b (with two major isoforms: 35 kDa and 24 kDa [1]) and LAPTM5. LAPTM4a and b are closely related with ∼43% amino acid sequence conservation, and more distantly related to LAPTM5 (∼23–25% conservation). Sequence homology is strong in the putative α-helical transmembrane segments and C-termini and suggests conserved function and/or sorting of these proteins [2], [3]. LAPTM4a and LAPTM4b are ubiquitously expressed, while LAPTM5 is expressed in immune cells [1], [3], [4], [5], [6], [7]. LAPTM5 has been implicated in regulation of B cell and T cell receptor surface expression, and is downregulated in multiple myeloma [8], [9], [10]. Interestingly, both LAPTM4 proteins play a role in multidrug resistance. When overexpressed in yeast, LAPTM4a confers multidrug resistance as a small molecule transporter capable of altering yeast sensitivity to small molecules, such as nucleosides and nucleobase analogs, antibiotics, anthracyclines, ionophores and steroid hormones [7]. LAPTM4b may mediate multidrug resistance by interacting with the multidrug resistance protein MDR1 [11], [12]. Moreover, LAPTM4b is overexpressed in various cancers [1], [3], [12], [13], [14], [15], [16], [17], [18], [19], [20], [21], [22] and has been implicated in the tumorigenic process [11], [21], [23], [24], [25], [26]. Overexpression of LAPTM4s may, therefore, enhance the proliferative and/or detoxification potential of cells, likely supporting their cancerous transformation or maintenance. Thus, identifying LAPTM4s sub-cellular localization and the factors that regulate their sorting could serve as the basis for strategies to counteract deleterious consequences of their overexpression.

All LAPTM proteins are assumed to localize to the late endosomes and lysosomes, which has been confirmed for LAPTM5 [4], [27] and LAPTM4a [5], [6]. LAPTMs contain several putative lysosomal targeting motifs in their C-termini, including tyrosine based (YXXΦ), PY (L/PPXY) and dileucine ([DE]XXXL[LI]) motifs. With the exception of PY motifs, these motifs are recognized by major adaptor molecules involved in trafficking from the Golgi to the lysosome (directly or indirectly via the plasma membrane) including the adaptor proteins AP1 to AP4 and GGA1-3 [28]. APs and GGAs bind cargo, protein coats and other accessory proteins for transport to and from specific compartments [29], [30]. Our lab recently demonstrated that lysosomal targeting of LAPTM5 is dependent on its C-terminal PY motifs, ubiquitin interacting motif (UIM) and an interaction with the E3 ubiquitin ligase Nedd4 (neuronal precursor-cell expressed developmentally down-regulated 4) [27]. Nedd4 proteins are comprised of a C2 domain, 3–4 WW domains that bind substrates by interacting with PY motifs, and a ubiquitin ligase HECT domain [31]. This mode of sorting is also used by the yeast vacuolar protein Sna3p [32]. Interestingly, both LAPTM4a and b contain PY motifs, but their role in targeting has not been investigated to date.

Here, we investigated the lysosomal and plasma membrane sorting of LAPTM4a and LAPTM4b, and show that their PY motifs, as well as Nedd4, participate in their subcellular sorting. We demonstrate that LAPTM4a and b, like LAPTM5, are present in the late endosomal and lysosomal compartments, but unlike other LAPTM members, LAPTM4b is also expressed at the plasma membrane, a site that is favoured upon mutation of its PY motifs.

## Results

### Cellular localization of LAPTM4 Proteins

**Figure 1:**
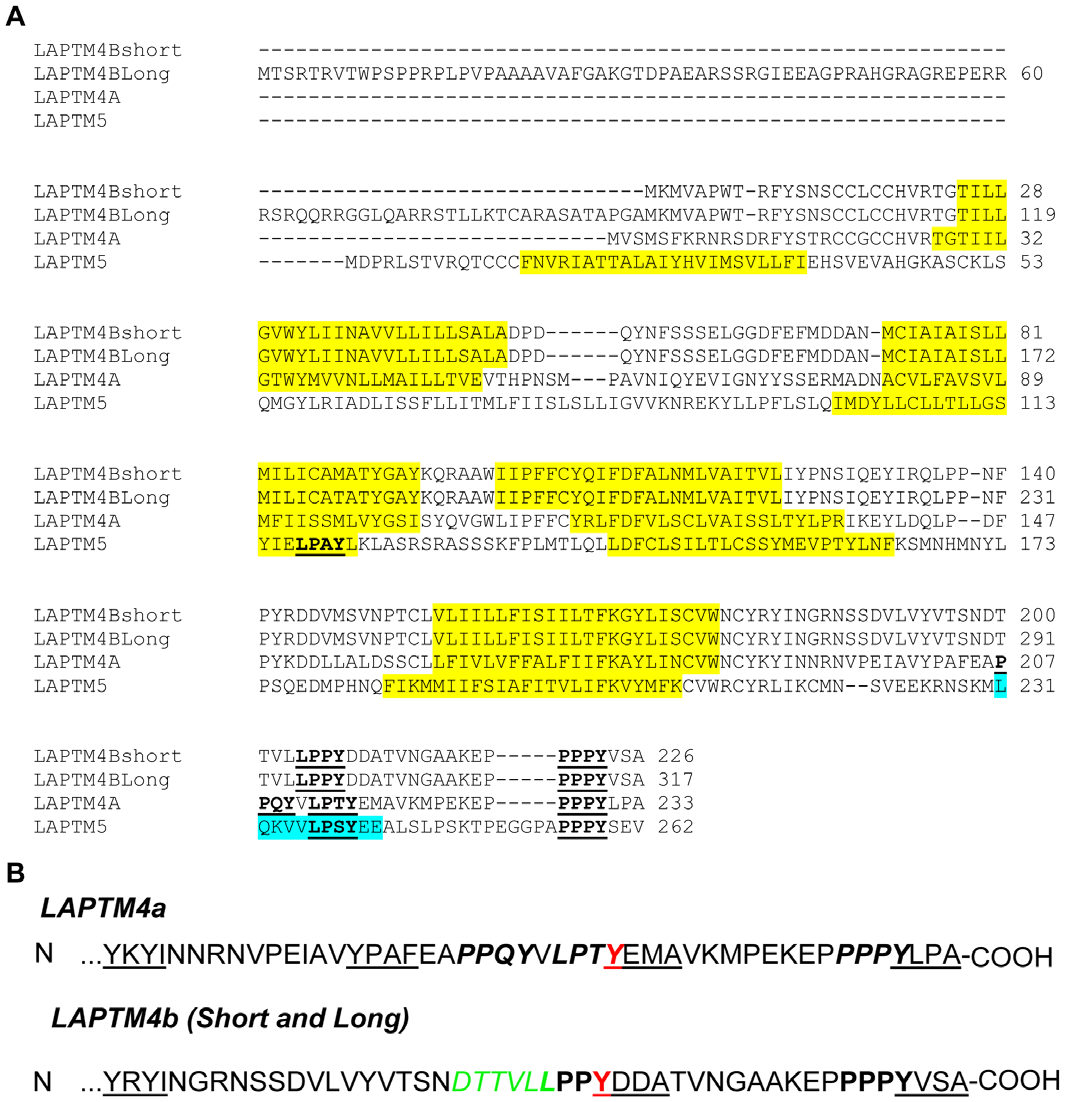
Sequence alignment of the LAPTM family of proteins. (A) ClustalW [49] amino acid sequence alignment of the LAPTM4 family members LAPTM4a (GenBank:AAH03158.1), 24 kDa-LAPTM4b (short) (GenBank: AAH31021.1), 35 kDa-LAPTM4b (long) (NP_060877.3) and LAPTM5 (GenBank: AAI06897.1). Putative transmembrane regions (predicted by SMART [50]) are highlighted in yellow, PY motifs are bold and underlined, and the UIM motif of LAPTM5 highlighted in blue. (B) PY motifs in the C-terminal tails of LAPTM4a and LAPTM4b are indicated in bold. Putative Tyrosine-based motifs are underlined. The dileucine motif is indicated in green (*italicized*), with the bold Leu shared with the PY motif. Tyrosine residues shared by tyrosine-based and PY motifs are indicated in red.

**Figure 2:**
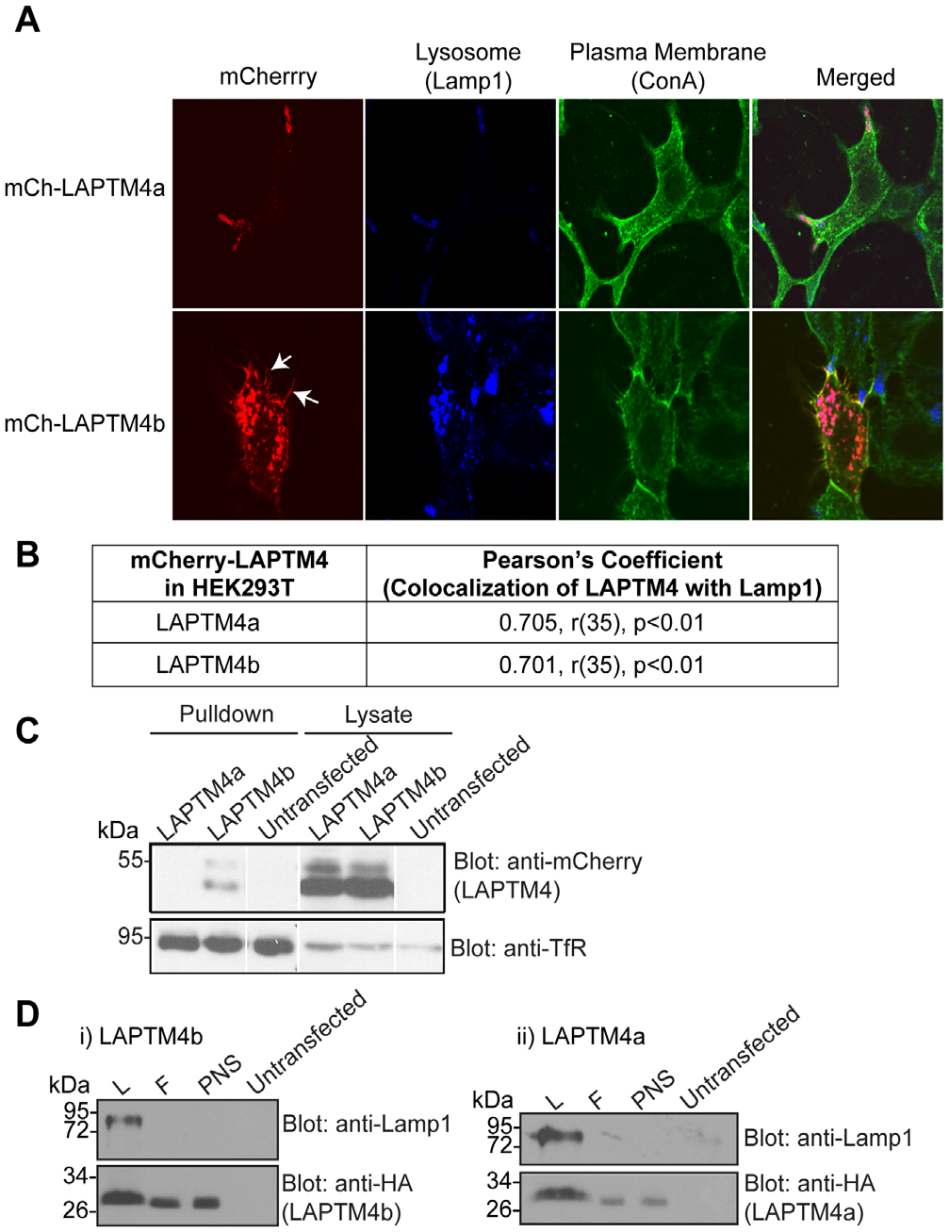
Both LAPTM4 proteins localize to lysosomes, but LAPTM4b is uniquely present also at the cell surface. (A) 24 hrs post transfection the plasma membrane (ConA, green) of Hek293T cells expressing mCh-LAPTM4a (red, top) or mCh-LAPTM4b (red, bottom) was stained, cells were fixed and incubated with anti-Lamp1 antibodies (blue). White arrows indicate mCh-LAPTM4b in hair-like protrusions. Cells were imaged using LSM510 and subcellular localization was assessed using Volocity 5.4.1. (B) Colocalization of LAPTM4s with the lysosomal marker Lamp1 was expressed in terms of the Pearson's correlation coefficient. Degrees of freedom are noted as (r), level of significance as (p). n = 37 for LAPTM4a and LAPTM4b. (C) Untransfected or transfected (mCh-LAPTM4a-WT or mCh-LAPTM4b-WT) Hek293T cells were subjected to cell surface biotinylation, lysis, pulldown with streptavidin agarose beads and separation on SDS-PAGE. Cell surface biotinylation of the Transferrin receptor (TfR) was used as a positive control. (D) Biochemical evidence for the presence of LAPTM4 proteins in lysosomes: Lysosomal fraction from Hek293T cells overexpressing HA-LAPTM4a or b were isolated as described in the Materials and Methods section. Post nuclear supernatant (PNS) was loaded on a magnetic column, the flowthrough (F) was collected and upon removal of the column from the magnet the lysosomal fraction (L) was eluted. Western blotting shows significant enrichment of endogenous Lamp1 in the L fraction, as well as the presence of LAPTM4a or b.

LAPTM4a and LAPTM4b are closely related family members (Figure 1). To detect sub-cellular localization of these two proteins, we generated mCherry-tagged (mCh) LAPTM4a and LAPTM4b (the 24 KDa isoform) constructs and expressed them in Hek293T cells (which endogenously express these proteins). Figure 2A,B shows that both LAPTM4a and LAPTM4b co-localized significantly with the late endosomal and lysosomal marker Lamp1. Unlike LAPTM4a, however, some LAPTM4b was also detected at the plasma membrane. In support, cell surface biotinylation experiments revealed the presence of LAPTM4b (but not LAPTM4a) at the cell surface (Figure 2C). These results suggest that a fraction of LAPTM4b is present at the plasma membrane. In order to biochemically confirm the presence of LAPTM4b in the lysosomal compartment, lysosomes were isolated from HA-LAPTM4b overexpressing cells using magnetic affinity chromatography, employing a previously described method [47], [48]. As expected, the lysosomal fraction isolated is enriched for endogenous Lamp1 relative to an equal amount of post-nuclear supernatant (PNS) input. Similar to Lamp1, HA-LAPTM4b was detected in the lysosomal fraction, as was LAPTM4a (Figure 2D).

**Figure 3:**
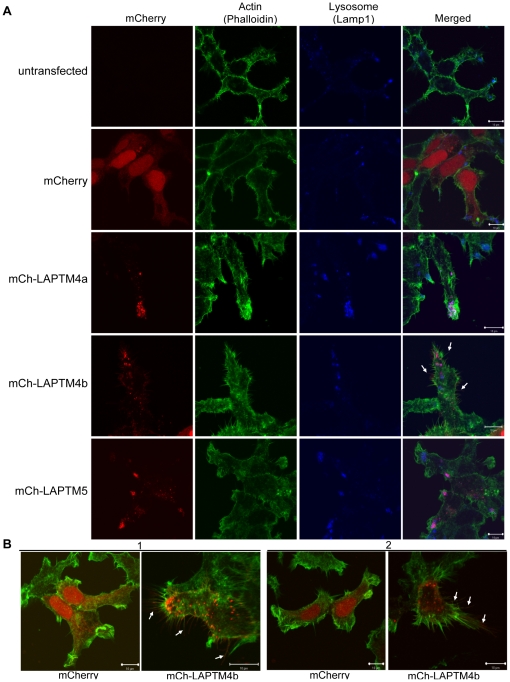
LAPTM4b, but not LAPTM4a or LAPTM5, is present in actin-based membrane protrusions in Hek293T cells. (A) Hek293T cells were transfected with mCherry alone, mCh-LAPTM4b-WT, mCh-LAPTM4a-WT or mCh-LAPTM5-WT (red). 24 hrs post transfection cells were fixed and stained with anti-Lamp1 antibody (blue) and Phalloidin (green). (B) Additional images illustrate the changes in actin-based membrane protrusions associated with mCh-LAPTM4b-WT expression (relative to mCherry-alone expressing cells) in two independent experiments (1 and 2). All images were taken with LSM510 and analyzed by Volocity 5.4.1 (Perkin Elmer). White arrows indicate examples of actin-based membrane protrusions in which mCh-LAPTM4b is present.

Interestingly, our results show that cells expressing LAPTM4b also exhibit hair-like membrane protrusions that largely co-stained with the plasma membrane marker Concanavalin A (ConA), (Figure 2A, Table 1, Figure S1). In addition, the nature of the protrusions was examined by staining Hek293T cells either untransfected or transfected with mCherry or mCh-LAPTM constructs with the actin-binding protein phalloidin (Figure 3). Untransfected, mCh-, LAPTM5 and LAPTM4a -expressing cells showed similar and minimal hair-like protrusions (Figure 3A). In contrast, mCh-LAPTM4b expressing cells on average had approximately twice as many and twice as long actin-based membrane protrusions relative to untransfected or mCh-transfected cells (Figure 3A,B and Table 2).

**Table 1:** mCh-LAPTM4b co-stains with membrane-protrusions when overexpressed in Hek293T cells.

		Cells in which LAPTM4b is PRESENT in actin-based membrane protrusions		Cells in which LAPTM4b is ABSENT from actin-based membrane protrusions	
Experiment	Total Number of Cells Observed	# cells	% of Total Cells	# cells	% of Total Cells
**1**	29	26	89.7	3	10.3
**2**	37	32	86.5	5	13.5

**Table 2:** Hek293T cells in which mCh-LAPTM4b is overexpressed have longer and higher density of actin-based membrane protrusions.

		Hek293T			
Protrusions Property	Experiment	Untransfected (n = 20)	mCh(n = 20)	mCh-LAPTM4b (n = 20)	Statistical Significance c
**Average length (µm)^a^**	1	1.963±0.104	2.267±0.189	3.505±0.092	p<0.0001
	2	1.545±0.086	1.775±0.122	3.383±0.165	p<0.0001
**Average density** **(# protrusions/ µm^2^)^b^**	1	0.027±0.003	0.024±0.003	0.0560±0.004	p<0.0001
	2	0.034±0.003	0.020±0.002	0.0648±0.008	p = 0.0024

a,b The average protrusion length/density is indicated ± SEM.

c A student t-test was performed to determine the statistical significance of the difference between average length/density of actin protrustions of mCh-LAPTM4b and untransfected cells.

### LAPTM4 proteins bind Nedd4

**Figure 4:**
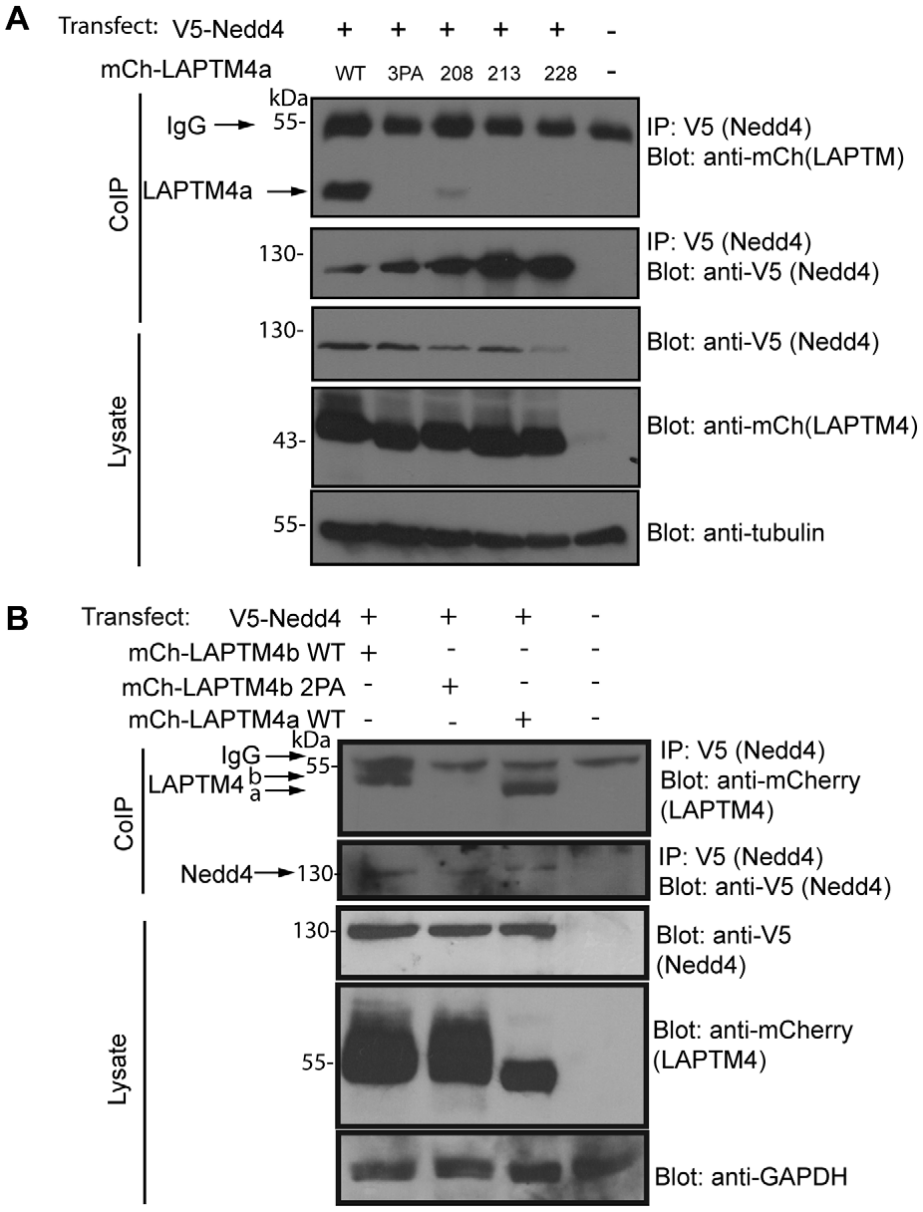
LAPTM4a and LAPTM4b bind Nedd4 via their PY motifs. (A) LAPTM4a's binding to Nedd4 is dependent on its 2nd and 3rd PY motifs. A Co-IP was performed on whole cell lysates from Hek293T cells co-expressing V5-tagged Nedd4 and mCh-LAPTM4a (WT, 3PA, P208A, P213A or P228A). Nedd4 was precipitated using anti-V5 antibodies, samples were separated on SDS-PAGE and transferred to nitrocellulose. Anti-mCherry antibodies were used to detect binding of mCh-LAPTM4s to Nedd4. (B) LAPTM4b binding to Nedd4 is dependent on its PY motifs. A CoIP was performed on whole cell lysates from Hek293T cells co-expressing V5-tagged Nedd4 and mCh-LAPTM4a-WT, mCh-LAPTM4b-WT or mCh-LAPTM4b-2PA, as in (A).The housekeeping proteins Tubulin and GAPDH were used interchangeably as loading controls for the cell lysates. (IgG bands are detected due to the HRP-conjugated 2^o^ antibody's specificity for mouse Ig, which detects the Ig heavy and light chains of the V5 antibody used during the pulldown.).

LAPTM5 was previously shown to interact with the E3 ubiquitin ligase Nedd4 via its C-terminal PY motifs [27]. Since PY motifs are also present in LAPTM4a and LAPTM4b (Figure 1B), we investigated their ability to bind Nedd4 in cells. Co-immunoprecipitation (co-IP) experiments were performed using cell lysates from Hek293T cells co-expressing V5-tagged Nedd4 and mCh-tagged LAPTM4a or LAPTM4b. Figure 4A and B show that both LAPTM4a and LAPTM4b were able to co-IP with Nedd4. Moreover, co-IP of LAPTM4a and LAPTM4b with Nedd4 was dependent on the presence of their PY motifs; Mutating all of LAPTM4a's 3 PY motif or LAPTM4b's 2 PY motif prolines to alanines (mCh-LAPTM4a-3PA or mCh-LAPTM4b-2PA) abolished binding to Nedd4 (Figure 4A, B). LAPTM4a's second (P213) and third (P228) PY motif appear most important for this interaction, as they are both unable to bind Nedd4 when mutated to Ala, while mutation of the 1^st^ PY motif (P208A) reduces, but does not eliminate, the binding of LAPTM4a to Nedd4 (Figure 4A). In addition, as with LAPTM5 [27] we show that Nedd4 can ubiquitinate both LAPTM4 proteins (Figure S2, Materials and Methods S1). Together, these experiments suggest that LAPTM4a and LAPTM4b bind Nedd4 in cells, and that this interaction is mediated via the LAPTM4a PY motifs.

### The PY motifs of LAPTM4 proteins regulate their sub-cellular distribution

**Figure 5:**
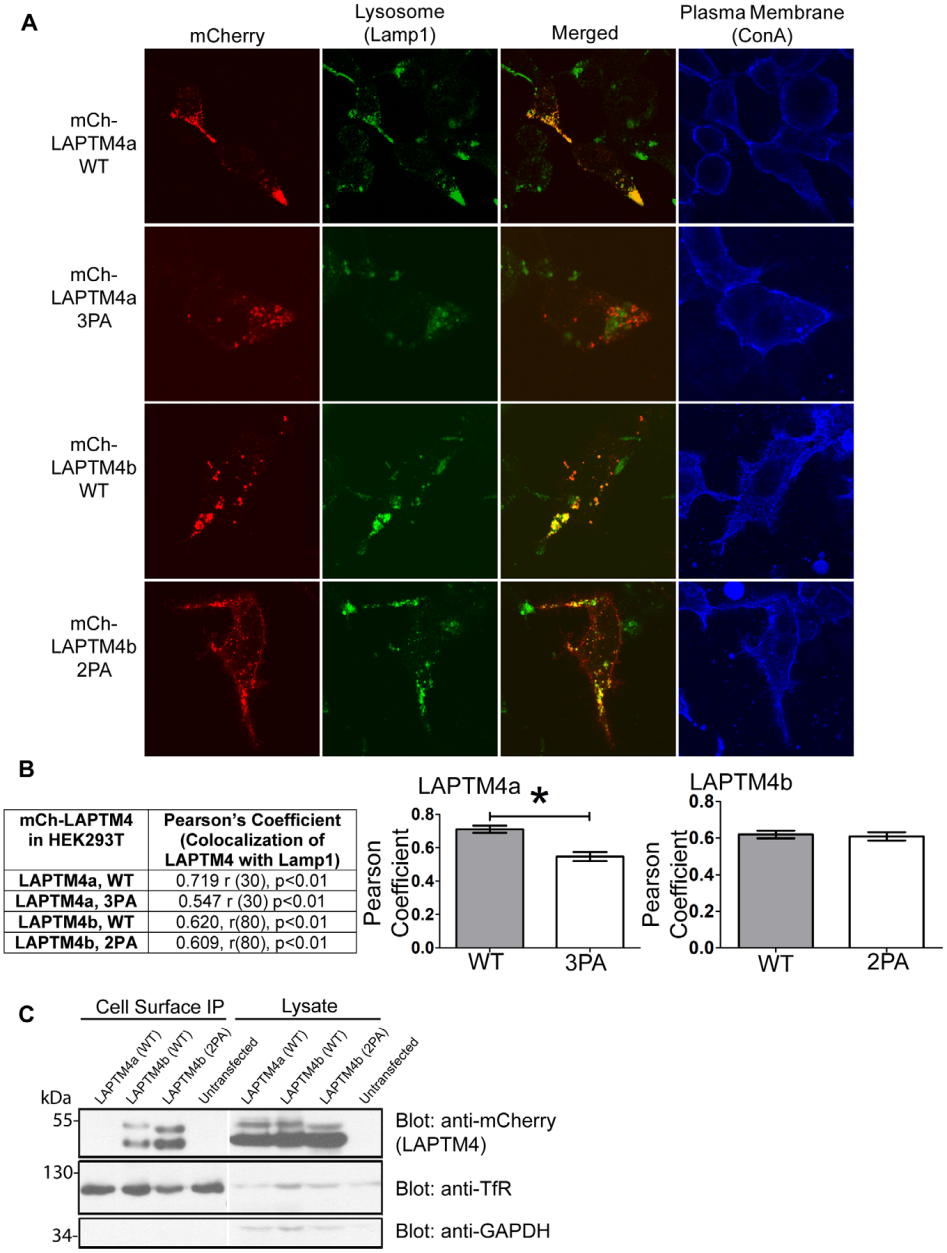
LAPTM4a and LAPTM4b require their PY motifs for proper targeting. (A) 24 hrs post transfection the plasma membrane of Hek293T cells expressing mCh-LAPTM4a-WT, mCh-LAPTM4a-3PA, mCh-LAPTM4b-WT or mCh-LAPTM4b-2PA was stained (ConA, blue), cells were fixed, incubated with anti-Lamp1 antibodies (green) and imaged by confocal microscopy. Subcellular localization of LAPTM4 was assessed using Volocity 5.4.1. (B) LAPTM4 colocalization with the lysosomal marker Lamp1 is expressed in terms of the Pearson's correlation coefficient. Degrees of freedom are noted as (r), level of significance as (p). Graphs illustrate changes in the mean Pearson's coefficients of mCh-LAPTM4 colocalization with Lamp1 (LAPTM4a n = 32, LAPTM4b n = 82). Error bars indicate SEM. * denotes p<0.0001. (C) Cell surface expression of mCh-LAPTM4b-2PA is up-regulated relative to that of mCh-LAPTM4b-WT. Cell surface biotinylation was performed on Hek293T cells expressing mCh-LAPTM4a-WT, mCh-LAPTM4b-WT or mCh-LAPTM4b-2PA. Cell surface biotinylation of Transferrin receptor (TfR) served as a positive control, while cell surface biotinylation of mCh-LAPTM4a-WT and GAPDH served as negative controls.

Because LAPTM4s interact with Nedd4 via their PY motifs and we previously showed that LAPTM5′s PY motifs are required for its proper localization to the lysosome in Hek293T and dendritic cells (DC) [27], we investigated whether the LAPTM4 PY motifs participate in their lysosomal targeting. Hek293T cells were transfected with WT or PY motif mutant LAPTM4a and LAPTM4b. At 24 hrs post transfection the subcellular localization of the LAPTM4 proteins was assessed by confocal microscopy. Figure 5 shows that while WT LAPTM4a and LAPTM4b both co-localized with the late endosome and lysosomal marker Lamp1, the PY motif deficient mCh-LAPTM4a-3PA shows significantly reduced co-localization (∼24% drop in colocalization). Interestingly mCh-LAPTM4b-2PA colocalization with Lamp1 was largely unchanged (∼2% drop in colocalization) (Figure 5B). This suggests that LAPTM4a and LAPTM4b differ in their dependence on the PY motifs for lysosomal sorting, with LAPTM4a being dependent and LAPTM4b minimally dependent on these motifs.

However, we noticed that while the mCh-LAPTM4b-2PA mutant was still able to localize to the lysosome, its surface expression appeared to have increased. In order to further investigate this observation, an experiment was conducted in which untransfected, mCh-LAPTM4a-WT, mCh-LAPTM4b-WT or mCh-LAPTM4b-2PA expressing Hek293T cells were subjected to cell-surface biotinylation. As seen in Figure 5C, there appears to be more mCh-LAPTM4b-2PA at the cell surface than mCh-LAPTM4b-WT. These data support the notion that the two PY motifs of LAPTM4b are important to minimize its missorting to the plasma membrane.

### Nedd4 facilitates the proper sorting of LAPTM4 proteins to the lysosome

**Figure 6:**
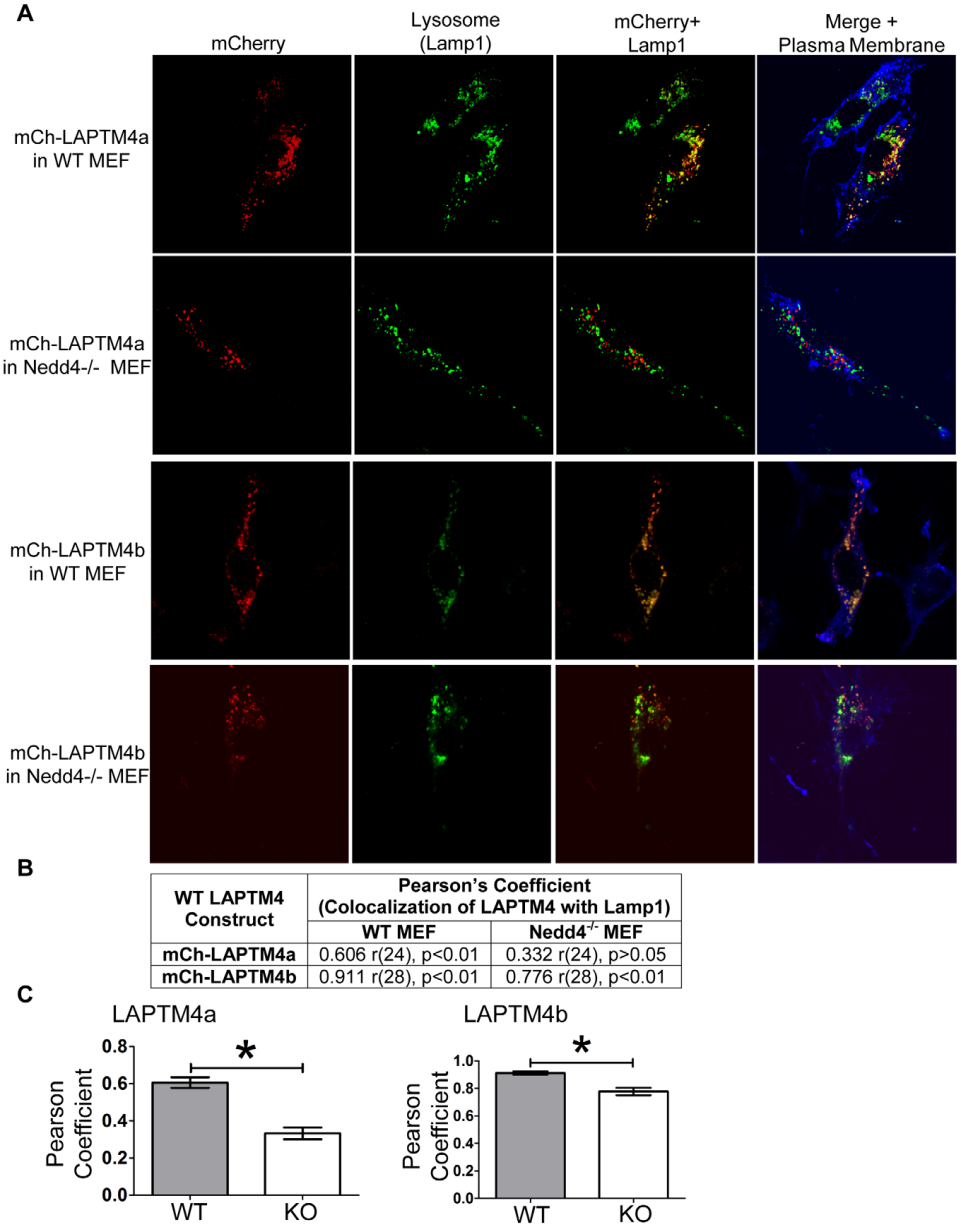
Nedd4 facilitates lysosomal sorting of LAPTM4a and LAPTM4b. (A) WT or Nedd4^−/−^ MEF cells were transfected with (wildtype) mCh-LAPTM4a or mCh-LAPTM4b (red). 24 hrs post transfection the plasma membranes was stained (ConA, blue), cells were fixed and incubated with anti-Lamp1 antibodies (green). Cells were imaged by confocal microscopy and subcellular localization was assessed using Volocity 5.4.1. (B) LAPTM4 colocalization with the lysosomal marker Lamp1 is expressed in terms of the Pearson's correlation coefficient. Degrees of freedom are noted as (r), level of significance as (p). (C) The graphs represent the changes in mean Pearson's coefficients of WT or Nedd4^−/−^ MEF cells expressing mCh-LAPTM4a (n = 26) or mCh-LAPTM4b (n = 30). Error bars indicate SEM and * indicates p<0.0001.

Given that all LAPTMs were able to efficiently co-IP with Nedd4 and Nedd4 is required for the proper localization of LAPTM5 to the lysosome [27], we investigated the role of Nedd4 in LAPTM4 lysosomal targeting using Nedd4 knockout (Nedd4^−/−^) MEFs that we recently generated [33]. WT or Nedd4^−/−^ MEF cells were transfected with mCh-LAPTM4a or mCh-LAPTM4b. After 24 hrs, the cells were fixed and stained with the lysosomal marker (Lamp1) and co-localization of the LAPTM4 protein was assessed using confocal microscopy (Figure 6). mCh-LAPTM4a expressed in WT MEFs showed strong colocalization with the lysosomal marker Lamp1 (Figure 6A). In contrast, LAPTM4a exhibited severely reduced (by 50%) lysosomal localization when expressed in Nedd4^−/−^ MEFs (Figure 6B,C). mCh-LAPTM4b expressing WT MEF cells showed some colocalization with the lysosomal marker Lamp1 and plasma membrane staining (Figure 6A). Quantification of the colocalization between mCh-LAPTM4b and Lamp1 in Nedd4^−/−^ and WT MEFs, revealed a decrease in colocalization of only ∼14%. This decrease is much smaller than that observed for LAPTM4a (Figure 6B,C), but is nonetheless significant. These results suggest that Nedd4 plays a significant role in the lysosomal sorting of LAPTM4a and a lesser, but still significant, role in that of LAPTM4b.

## Discussion

Our studies here provide evidence (i) that LAPTM4b, like LAPTM4a and LAPTM5, localizes to lysosomes, (ii) that both LAPTM4a and LAPTM4b can co-IP with the ubiquitin ligase Nedd4 and can be ubiquitinated by it, albeit at different strengths, and (iii) that sorting of LAPTM4a (and to a lesser extent LAPTM4b) is dependent on interaction with Nedd4, similar to our previous observation with LAPTM5. However, despite these commonalities, we also show that LAPTM targeting is not entirely conserved: LAPTM4b, unlike other LAPTMs, also localizes to the plasma membrane (and appears to enhance the formation of actin-based membrane protrusions) and its dependence on Nedd4 for lysosomal sorting is weaker than that of LAPTM4a. Moreover, LAPTM4b sorting to the plasma membrane is enhanced upon of loss of its PY motifs, suggesting that Nedd4 normally prevents it from sorting to the cell surface.

Our current studies, along with our (and others') earlier work, indicate that all LAPTM proteins reside within the endo-lysosomal system. While localization was previously shown for LAPTM5 [4], [27] and LAPTM4a [5], [6], LAPTM4b localization had been assumed to be lysosomal from its association with membrane fractions and immunohistochemical staining of cancerous tissues [1], [18], [19], [21], [34]. The lysosomal localization of LAPTM4 proteins supports their putative roles in small molecule transport ([7], [11], [12] and Milkereit et al. (unpublished data)), as sequestration of bioactive compounds to this organelle precludes their activity [6], [7], [12]. The detection of LAPTM4b at the plasma membrane is in accord with the observation that it is able to bind to MDR1 (a plasma membrane expressed multidrug transporter) [11] and agrees with the expression pattern of other multidrug transporters which are found simultaneously at the plasma membrane and internal organelles (Golgi/Endosomes/Lysosomes) [35], [36]. Since LAPTM4b is the only LAPTM member present at the cell surface, it likely has some unique functions. One such unique function may include the formation of membrane protrusions. The actin-based membrane protrusions induced by LAPTM4b are reminiscent of filopodia associated with the epithelial-mesenchymal transition (EMT) and metastasis of cancerous cells [37], [38], [39]. It is possible that filopodia growth contributes to the increased invasive potential and metastasis associated with LAPTM4b overexpressing cancers and cells [21], [25], [26]. While the membrane protrusions we observed were likely enhanced by LAPTM4b overexpression, such overexpression is common in numerous cancers and likely contributes to the metastatic potential of LAPTM4b -expressing cells, as described above.

We have shown that LAPTM4 proteins, like LAPTM5, coimmunoprecipitates with Nedd4. We believe that this interaction is most likely direct, as this co-IP was abolished by mutating the LAPTM4 PY motifs. While we cannot currently prove that mutation of the PY motifs did not cause other alteration in the LAPTM4 proteins that resulted in loss of binding to Nedd4, the PY motif mutants appear to be expressed at the right size and appear stable, suggesting that they are not misfolded.

Our localization studies suggest Nedd4 plays differing roles for each LAPTM member. In the case of LAPTM5, we previously established that the loss of Nedd4 or functional PY motifs, resulted in LAPTM5 Golgi retention [27]. This stands in contrast with the phenotype observed for LAPTM4s: in the absence of PY motifs or Nedd4 LAPTM4a exits the Golgi, but co-localization with Lamp1 positive vesicles decreases (∼50%). Similarly, the loss of PY motifs in LAPTM4b, or lack of Nedd4, does not affect Golgi exit and only partially affects lysosomal localization. Instead, the absence of PY motifs enhances the proportion of LAPTM4b at the cell surface. The role of Nedd4 in regulating lysosomal versus plasma membrane sorting described here is reminiscent of nutrient-dependent sorting of Gap1 permease in yeast, which is controlled via Gap1 ubiquitination by Rsp5, the *S. cerevisae* orthologue of Nedd4 [40], [41]. In addition, while LAPTM5 possesses a UIM motif that is also necessary for its lysosomal sorting (by interacting with ubiquitinated GGA3) [27], LAPTM4 proteins lack UIM motifs. In accord, our preliminary data show that unlike LAPTM5 [27], LAPTM4a cannot bind ubiquitin. Thus, despite sequence similarities between the LAPTM family members, the regulation of their sub-cellular localization is not identical (Figure S3).

In fact, the greater the LAPTM4 sequence differs from that of LAPTM5, the smaller the role of Nedd4 appears to be in its sorting. This relationship may be secondary to the number and context of LAPTM4 PY motifs. Like LAPTM5, LAPTM4a contains three PY motifs, whereas LAPTM4b contains only two. While a single PY motif is sufficient to bind a WW domain, it is possible that having numerous PY motifs could enhance binding avidity. Furthermore, the context of the PY motifs may affect Nedd4's ability to mediate targeting. Based on Figure 1B, one could speculate that other LAPTM4 C-terminal putative lysosomal targeting motifs, in addition or in combination with PY motifs, could play a role in LAPTM4 lysosomal targeting. Should additional motifs play a role, access to PY motifs could be spatiotemporally controlled. For instance, if a tyrosine-based motif or a dileucine motif has a higher affinity for their binding partner than a neighboring or overlapping PY motif, the PY motifs may become obscured. This is particularly relevant when considering the LAPTM4b PY motifs, which share residues not only with a potential dileucine targeting motif, but also a putative tyrosine based motif. Indeed, two tandemly arranged tyrosine-based (YXXΦ) motifs were previously proposed to be involved in LAPTM4a lysosomal sorting[2]. However, the substitution mutation made to the tyrosine of the 2^nd^ YXXΦ motif simultaneously disrupted a PY motif (Figure 1B) raising the possibility that a disrupted PY motif may have contributed (wholly or in part) to the observed missorting. Nonetheless, involvement of additional putative lysosomal targeting motifs may explain why ∼50% and 86% of LAPTM4a and LAPTM4b, respectively, were correctly sorted to lysosomes, despite the absence of Nedd4 in our MEFs. Having numerous lysosomal targeting motifs may mediate efficient lysosomal delivery even if one or more lysosomal targeting pathways are impaired [42], [43]. Such a mechanism for LAPTMs remains speculative, but would be relevant considering the potential consequences of LAPTM4 mislocalization with respect to small molecule transport [7], [11], [12] and/or cell signalling [11], [21], [23], [24], [26].

Additionally, while ubiquitination of LAPTM4b occurs in the presence ectopically expressed Nedd4, and is reduced in the presence of a catalytically inactive Nedd4(CS)(Figure S2, Materials and Methods S1), LAPTM4b is also ubiquitinated in Hek293Ts that are not transfected with Nedd4. This suggests that either endogenous Nedd4 (expressed in these cells) or/and other E3 ligases, is/are responsible for this ubiquitination. Potential other E3s may include additional Nedd4 family members, which also possess WW domains capable of binding PY motifs [31]. If other Nedd4 family members bind to and/or ubiquitinate LAPTM4b, this could also contribute to the different localization patterns observed when WT LAPTM4b is expressed in Nedd4^−/−^ MEFs (Figure 6A) and LAPTM4b-2PA in Hek293Ts (Figure 5A). While the absence of PY motifs results in an accumulation of LAPTM4b at the cell surface without altering lysosomal localization, the absence of Nedd4 leads to a minor decrease in LAPTM4b lysosomal co-localization without affecting its membrane expression. In such a scenario, we speculate that a LAPTM4b PY motif mutant might be unable to bind any Nedd4 family member via their WW domains. Other alternative motifs could then target LAPTM4b to the lysosome and play a role in the accumulation of LAPTM4b at the plasma membrane. In the absence of Nedd4 in the Nedd4^−/−^ MEFs, other Nedd4 family members could potentially assist in the clearance of LAPTM4b from the plasma membrane.

In conclusion, our results show Nedd4 and PY motifs are involved in LAPTM4 sorting. We believe that PY motifs may play a role in the sorting of additional lysosomal proteins. Of the 215 integral membrane proteins identified in rat liver tritosomes (lysosomes) [44], we identified 20 that possess PY motifs in humans (Table S1, Materials and Methods S1). Whether any of these is/are also regulated by Nedd4 family members remains to be established.

## Materials and Methods

### Cell Lines, Antibodies and Reagents

Hek293T cells (Obtained from ATCC) were cultured in DMEM supplemented with 10% fetal bovine serum (FBS), 100 U/ml penicillin, and 100 µg/ml streptomycin. Cells were transfected using the calcium phosphate transfection method[45]. Mouse embryonic fibroblasts (MEF) (WT and Nedd4^−/−^ MEF) were generated as described earlier[33]. MEF cells were cultured in DMEM supplemented with 10% FBS, 100 U/ml penicillin, 100 µg/ml streptomycin and 100 µg/ml glutamine. They were transfected according to the manufacturer's protocol using ESCRTV transfection reagent (Sigma-Aldrich). For confocal imaging: Mouse monoclonal [H4A3] to Lamp1 (Abcam), rat anti-mouse Lamp1 **(**BD Pharmingen**)**, Normal Goat Serum (Jackson ImmunoResearch Laboratories Inc.), Alexa-Fluor 647 conjugated ConcanavillinA (ConA, Invitrogen), Alexa-Fluor 488 Conjugated Phalloidin (Invitrogen), Alexa-Fluor 488 Goat-anti mouse 2^o^ antibody (Invitrogen), Alexa-Fluor 488 Goat-anti rat 2^o^ antibody (Invitrogen). Slides were mounted using Darkocytomation (Dako Corporation). For Immunoprecipitation and Western Blotting: Peroxidase-conjugated AffiniPure Goat Anti-Mouse IgG(H+L) (Jackson ImmunoResearch laboratories Inc.), Living Colors mCherry Monoclonal Antibody (Clontech), GAPDH (A-3, Santa Cruz), anti-Human Transferrin receptor antibody #13-6800 **(**Zymed), anti-Tubulin (Sigma), anti-β-actin (Sigma), anti-Hemagglutinin (HA, Covance), anti-V5 (AbDserotec), anti-Penta-His (Invitrogen), anti-Lamp1 (Abcam). Protein-G Agarose (BioShop Canada), Streptavidin Agarose (Thermo Scientific). Nickel-NTA Agarose Beads (Qiagen). LLnL (N-acetyl-Leu-Leu-norleucinal, Sigma).For Biotinylation: EZ-link Sulfo-NHS-SS-biotin (Thermo Scientific). Developed with Western Lightning™Plus-enhanced chemiluminescence (ECL,Perkin Elmer). miniMACS columns for magnetic affinity chromatography (Miltenyi Biotec Inc.).

### Constructs

We obtained cDNA entry clones of human LAPTM4a (GenBank: AAH03158.1) and LAPTM4b (the 24 kDa isoform, GenBank: AAH31021.1) from Invitrogen, and used these cDNAs in all our experiments. Site directed mutagenesis was used to mutate the 2^nd^ conserved Proline in the PY-motifs (L/PPXY) to Alanine. LAPTM4a single PY-motif mutants are P208A, P213A, P228A, while the total PY motif mutant is LAPTM4a-3PA. The total PY motif mutant of LAPTM4b is LAPTM4b-2PA (mutated residues are P205A and P221A). WT and PY motif mutants were N-terminally tagged with mCherry or HA (Hemagglutinin) using the Gateway cloning system (Invitrogen). The histidine-tagged ubiquitin construct (His-Ub) was provided by D. Bohmann (University of Rochester Medical Center, Rochester, NY;[46]).

### Immunofluorescent Confocal Microscopy

Hek293T and MEF cells were cultured on poly-D-Lysine coated coverslips in 6-well-plates. 24 hrs post transfection wells were washed 3x with 1 ml PBS and incubated 5 min with Alexa-Fluor-647-conjugated ConA (1∶1000). After three PBS washes, cells were fixed with 4% Paraformaldehyde (PFA), permeabilized with 0.1% Triton X-100 and incubated with 1∶100 Normal Goat Serum in 3% Skim Milk (30 min). Slides were incubated 1 hr with mouse anti-human (Hek293T) or rat anti-mouse (MEFs) Lamp1 (1∶1000) in 3% Skim milk. After three PBS washes, cells were incubated with Goat anti-mouse or anti-rat Alexa 488 Fluor-conjugated antibody. When applicable, Phalloidin-staining (1∶1000) was performed concurrently with the 2^0^ antibody. Cover slips were mounted with Dako Cytomation and imaged with a LSM510 confocal microscope at 63x magnification with a 1.4NA oil-immersion objective (Carl Zeiss MicoImaging, Inc.). Colocalization of Lamp1 and mCh-tagged LAPTM4s was assessed by Volocity 5.4.1 (Perkin Elmer) and expressed in terms of the Pearson's correlation coefficients.

### Characterization of the Actin-based Membrane Protrusions

Hek293T cells (untransfected, or transfected with mCh- or mCh-LAPTM4b-WT) were stained with phalloidin as described above. The length of actin-based membrane protrusions was determined using the line tool in Volocity 5.4.1 (Perkin Elmer). Each phalloidin-stained actin-based membrane protrusion was measured per cell and the average length of the protrusions was determined. The average density of protrusions per µm^2^ was determined by computing the total number of actin-based membrane protrusions per cell divided by the total cell's surface area. The average length and density of the protrusions indicated per condition (untransfected, mCh- or mCh-LAPTM4b-WT) in Table 2 represent the average length and density of actin-based membrane protrusions of 20 cells.

### Co-Immunoprecipitation (Co-IP) Assays

Hek293T cells were co-transfected with mCh-tagged LAPTM4s and V5-tagged Nedd4. 24 hrs post transfection media was changed. At 48 hrs, cells were lysed on ice in 1 ml Lysis buffer (50 mM Hepes, pH 7.5, 150 mN NaCl, 1% Triton X-100, 10% glycerol, 1.5 mM MgCl_2_, 1.0 mM EGTA, 10 µg/ml leupeptin, 10 µg /ml aprotinin, 10 µg /ml pepstatin, 1 mM PMSF) and centrifuged at 20817 rcf (g) (30 min, 4°C). 1 mg of lysate was incubated with 1.5 µl anti-V5 antibody and 15 µl Protein-G Sepharose beads (4°C, 4 hrs). Tubes were spun at 425 rcf (g) (3 min), washed 3x with Lysis Buffer and 3x with low salt HNTG (20 mM Hepes, pH 7.5, 150 mM NaCl, 10% glycerol, and 0.1% TritonX-100). Proteins were eluted with 30 µl 1xSDS-PAGE sample buffer, resolved on 15% SDS-Poly-acrylamide Gels (SDS-PAGE) and transferred to nitrocellulose. Upon cell lysis, 50 µg of fresh cell lysate are set aside and loaded on the same 15% SDS-PAGE gel as “Lysate.” This serves as a control to confirm and monitor the expression of the transfected proteins. For Western blotting: the membrane was blocked in 3% Dry Milk (30 min), incubated 1 hr with primary antibody (anti-mCh 1∶2500; anti-V5 1∶5000; anti-GAPDH 1∶1000, anti-Tubulin 1∶1000), washed 3x with wash buffer (PBS, 0.1% Triton X-100), incubated with Horseradish peroxidase conjugated Goat anti-mouse IgG (45 min) and developed using enhanced chemiluminescence (ECL). All centrifugations were performed using an Eppendorf Centrifuge 5417R.

### Cell Surface Biotinylation Assay

Hek293T were transfected with mCh-LAPTM4a-WT, mCh-LAPTM4b-WT or mCh-LAPTM4b-2PA. At 24 hrs post-transfection cells were washed 3x with PBS and incubated with 3 ml of 1 mg/ml EZ-link Sulfo-NHS-SS-biotin in PBS (1 hr, 4°C). Cells were washed 3x in PBS and lysed in 1 mL Lysis Buffer. Lysates were collected and centrifuged at 20817 rcf(g) (30 min, 4°C). Upon cell lysis, 50 µg of fresh cell lysate are set aside and loaded on the same SDS-PAGE gel as “Lysate.” This serves as a control to confirm and monitor the expression of the transfected proteins. 1 mg of supernatant was incubated with 30 µl Streptavidin agarose beads (4 hrs, 4°C). Beads were spun at 956 rcf (g) (2 min), washed 3x with 1 ml Lysis Buffer and 3x 1 ml Low Salt HNTG. Biotinylated proteins were eluted in 30 µL 1xSDS-PAGE sample buffer (5 min, 100°C). Samples were resolved by 15% SDS-PAGE and transferred to nitrocellulose. Western blotting: membrane was blocked in 3% Dry Milk (30 minutes), incubated with anti-mCherry (1∶2500); anti-Transferrin (1∶1000) or anti-GAPDH (1∶1000) for 1 hr. Blots were washed 3x with wash buffer, incubated with Horseradish peroxidase conjugated Goat anti-mouse IgG (45 min) and were developed with ECL.

### Isolation of Lysosomes using Magnetic Affinity Chromatography

Lysosomes were isolated as previously described by magnetic affinity chromatography using superparamagnetic (Iron-Dextran (FeDex)) particles [47], [48]. Briefly, Hek293T in 20 cm dishes were transfected with 20 µg HA-LAPTM4a or b. At 24 hrs the cells were incubated with 20 ml colloidal iron dextran particles in 20 ml lyophilized media. After 9 hrs, cells were washed 3x with PBS and chased with fresh media overnight. Cells were washed 1x with PBS, trypsinized, collected in 5 mL fresh media and spun at 956 rcf (g) for 5 min. Lysosomes were isolated at 4°C. The pellet was resuspended in 40 ml homogenization buffer (0.25 M sucrose, 4 mM imidazole, pH 7.4, 10 µg/ml leupeptin, 10 µg/ml aprotinin, 10 µg/ml pepstatin, 1 mM PMSF) and spun at 956 rcf (g) for 5 minutes. The pellet was homogenized in 2 ml homogenization buffer and spun for 10 min. at 956 rcf (g). 1 ml post-nuclear supernatant (PNS) was collected and loaded onto a miniMACs column on a magnet. The flowthrough (F) was collected and the column was washed 2x with homogenization buffer. The column was removed from the magnet and the lysosomal fraction was eluted with 300 µl Elution Buffer (5 mM Tris pH 7.4, 150 mM NaCl, 0.1% Triton). 10 µg of untransfected Hek293T cell lystate, PNS, Flowthrough (F), and Lysosomal Fraction (L) were loaded on a 15% SDS-PAGE and transferred to nitrocellulose. A western blot was performed as described for Co-IP experiments, with anti-Lamp1 (1∶1000) or (1∶10 000) anti-HA antibodies.

## Supporting Information

Figure S1 **mCh-LAPTM4b co-stains with plasma membrane protrusions.** Three view fields selected at random of Hek293T cells (Table 1) expressing mCh-LAPTM4b (red) at 24 hrs post transfection are shown. The plasma membrane is stained with ConA (green). Cells were imaged using LSM510. The brightness of the red channel has been increased approximately 2 fold using Volocity 5.4.1.
(TIF)

Figure S2 **Nedd4 ubiquitinates LAPTM4a and LAPTM4b in cells.** (A) LAPTM4a is ubiquitinated by Nedd4. Hek293T cells co-expressing V5-tagged Nedd4 (WT or catalytically inactive CS mutant) and HA-LAPTM4a (WT or 3PA) and His-Ub were lysed, lysate boiled in SDS to dissociate putative interacting proteins, and diluted (see Methods). Proteins tagged by His-Ub were precipitated using Ni-NTA agarose beads, samples were separated on SDS-PAGE and transferred to nitrocellulose. Anti-HA antibodies were used to detect His-Ubiquination of LAPTM4a. (B) LAPTM4b is ubiquitinated by Nedd4. As in (A), except using WT or 2PA HA-LAPTM4b. Actin is used as a housekeeping protein for the lysate loading control.
(TIF)

Figure S3 **Schematic representation of the contribution of Nedd4 to lysosomal sorting of LAPTM proteins (tested in Hek293T cells), depicting varying dependency on Nedd4, with LAPTM5 being highly-dependent (and also collaborating with GGA3), LAPTM4a exhibiting intermediate dependency (∼50%), and LAPTM4b minimally dependent on Nedd4.** ? represents other (unknown) factors.
(TIF)

Table S1 **Examination of the 215 peptide sequences of proteins identified as resident lysosomal integral membrane proteins in a screen of rat liver tritosomes** [44] **, has identified 20 proteins with conserved PY motifs in rat and human proteins using DNAassist (v3.0, University of the Free State).**
(DOC)

Materials and Methods S1 **Ubiquitination Assay.** Identification of PY Motif containing lysosomal proteins.
(DOC)
