# Supplementary figures and images for: A Role for the Ubiquitin Ligase Nedd4 in Membrane Sorting of LAPTM4 Proteins
Source: PLoS One. 2011 Nov 11;6(11):e27478. doi: 10.1371/journal.pone.0027478 (PMC3214061; doi:10.1371/journal.pone.0027478)

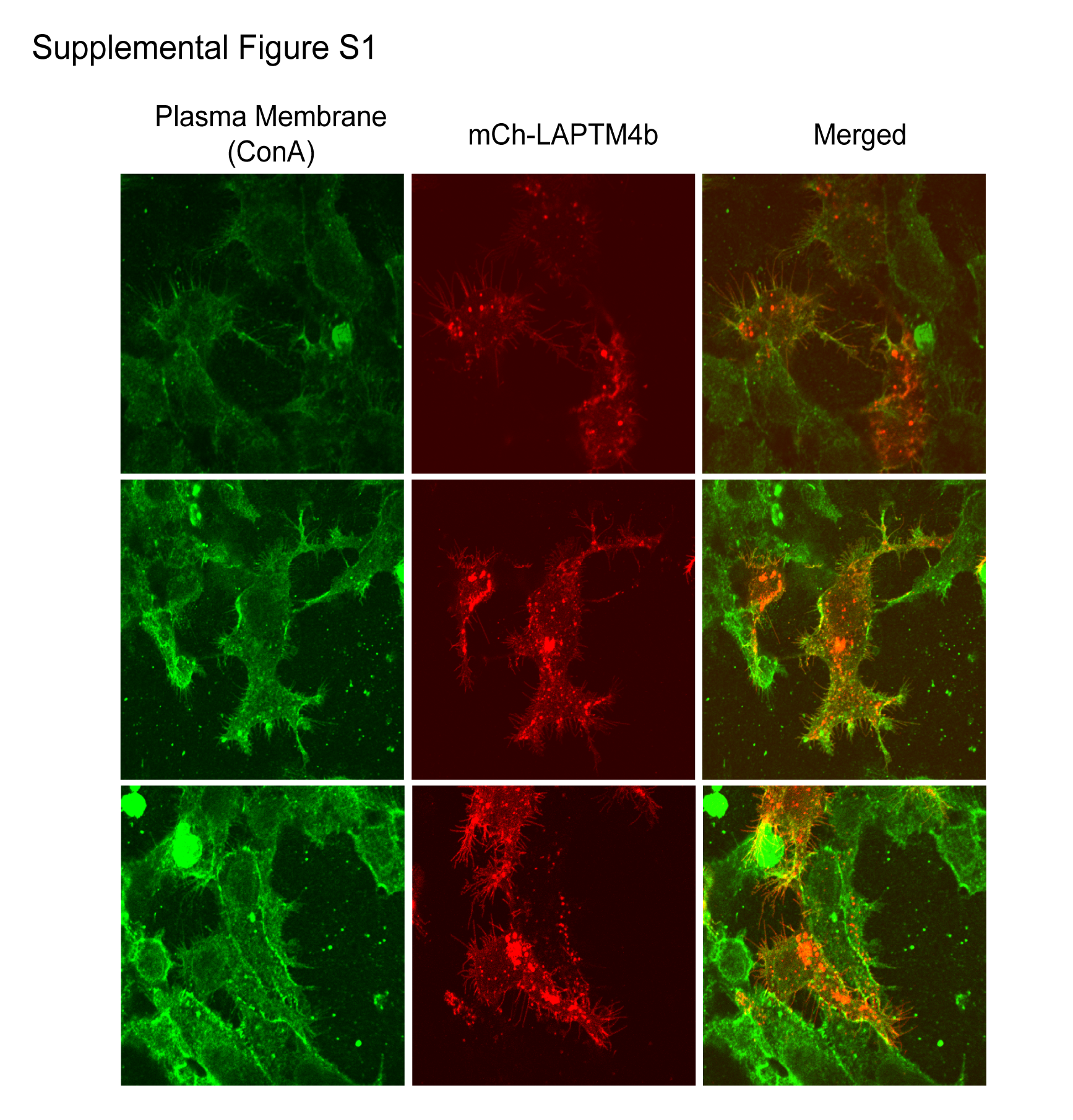

Supplement: Figure S1 — mCh-LAPTM4b co-stains with plasma membrane protrusions. Three view fields selected at random of Hek293T cells (Table 1) expressing mCh-LAPTM4b (red) at 24 hrs post transfection are shown. The plasma membrane is stained with ConA (green). Cells were imaged using LSM510. The brightness of the red channel has been increased approximately 2 fold using Volocity 5.4.1. (TIF) [file pone.0027478.s001.tif]

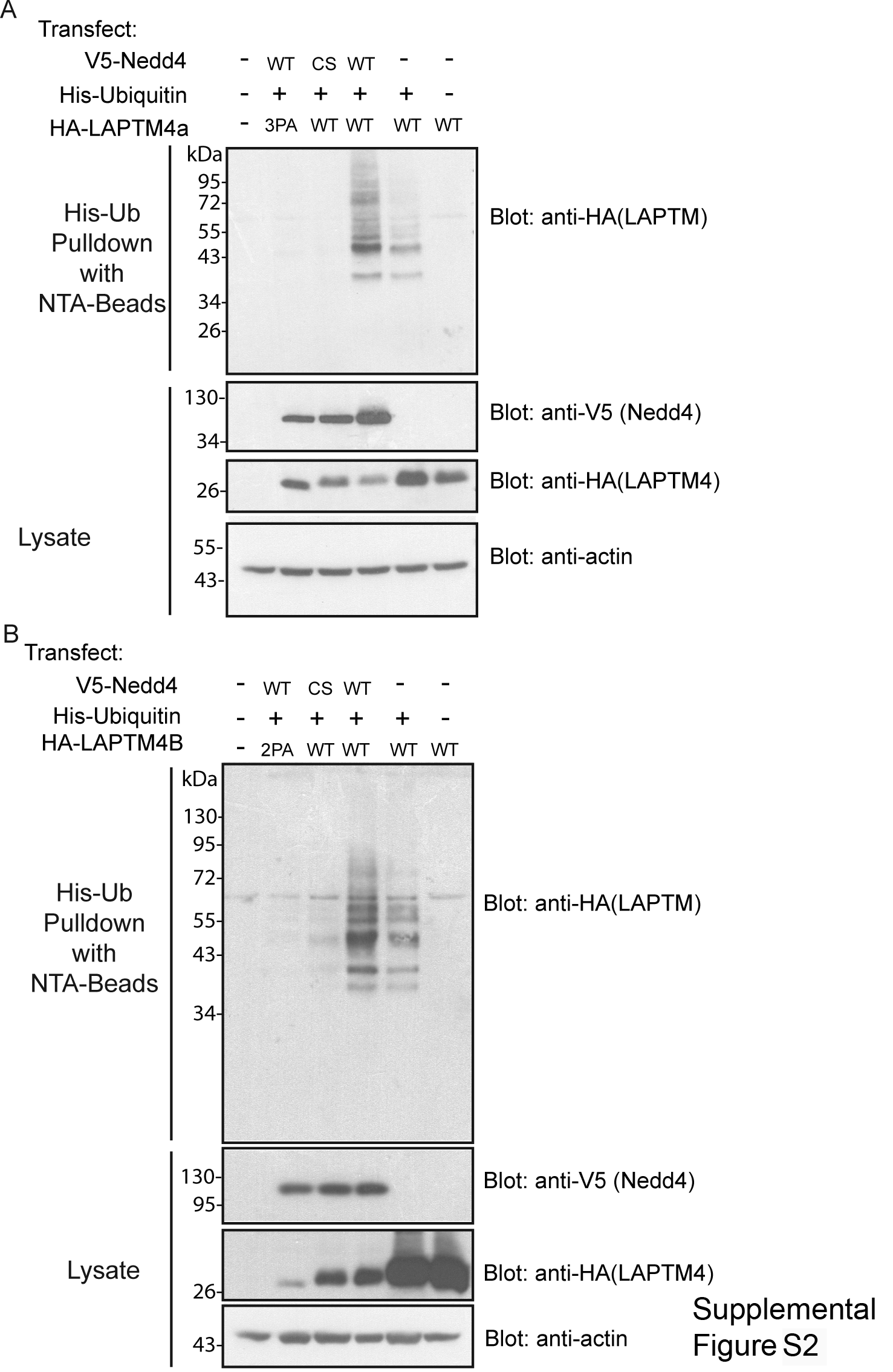

Supplement: Figure S2 — Nedd4 ubiquitinates LAPTM4a and LAPTM4b in cells. (A) LAPTM4a is ubiquitinated by Nedd4. Hek293T cells co-expressing V5-tagged Nedd4 (WT or catalytically inactive CS mutant) and HA-LAPTM4a (WT or 3PA) and His-Ub were lysed, lysate boiled in SDS to dissociate putative interacting proteins, and diluted (see Methods). Proteins tagged by His-Ub were precipitated using Ni-NTA agarose beads, samples were separated on SDS-PAGE and transferred to nitrocellulose. Anti-HA antibodies were used to detect His-Ubiquination of LAPTM4a. (B) LAPTM4b is ubiquitinated by Nedd4. As in (A), except using WT or 2PA HA-LAPTM4b. Actin is used as a housekeeping protein for the lysate loading control. (TIF) [file pone.0027478.s002.tif]

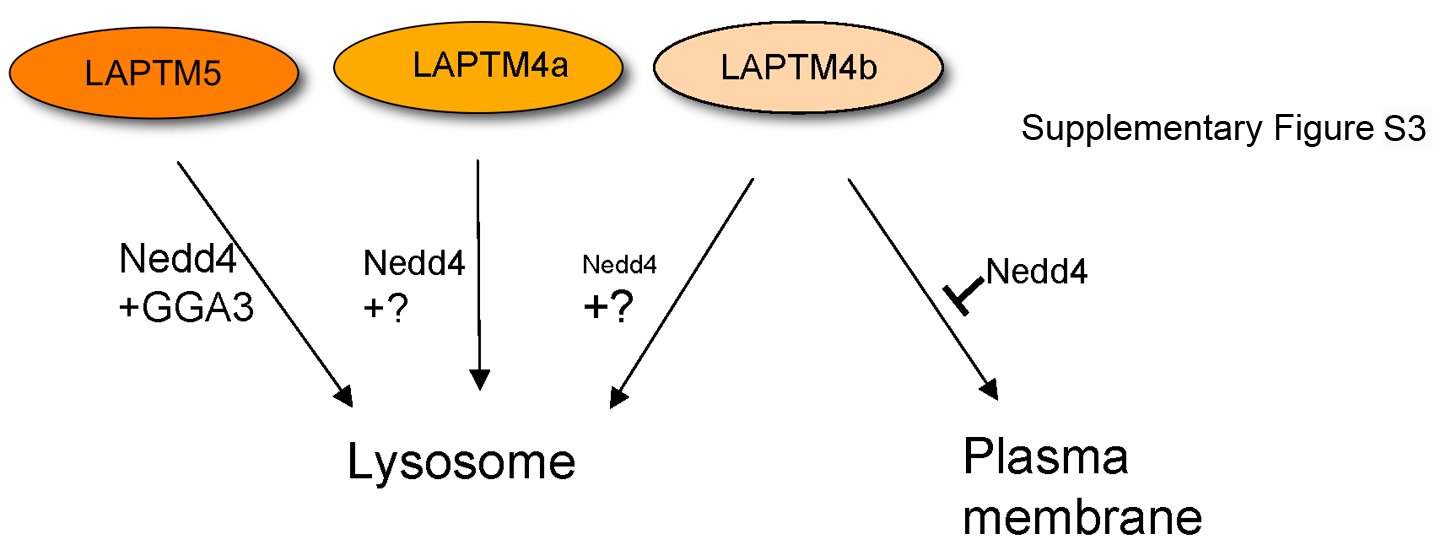

Supplement: Figure S3 — Schematic representation of the contribution of Nedd4 to lysosomal sorting of LAPTM proteins (tested in Hek293T cells), depicting varying dependency on Nedd4, with LAPTM5 being highly-dependent (and also collaborating with GGA3), LAPTM4a exhibiting intermediate dependency (∼50%), and LAPTM4b minimally dependent on Nedd4. ? represents other (unknown) factors. (TIF) [file pone.0027478.s003.tif]
