# Supplementary material for: A Role for the Ubiquitin Ligase Nedd4 in Membrane Sorting of LAPTM4 Proteins
Source: PLoS One. 2011 Nov 11;6(11):e27478. doi: 10.1371/journal.pone.0027478 (PMC3214061; doi:10.1371/journal.pone.0027478)
Supplement: Table S1 — Examination of the 215 peptide sequences of proteins identified as resident lysosomal integral membrane proteins in a screen of rat liver tritosomes [44] , has identified 20 proteins with conserved PY motifs in rat and human proteins using DNAassist (v3.0, University of the Free State). (DOC) [file pone.0027478.s004.doc]

**Table S1:** PY motifs are present in other lysosomal membrane proteins.

| **Protein Name** | **Accession Number** | **Putative PY Motif** |
| --- | --- | --- |
| UDP glycosyltransferase 2 family,polypeptide B | 13928718 | ppsy, lpwy |
| UDP-glucuronosyltransferase 2 family,member 5 | 34876712 | ppsy, lpwy |
| Niemann Pick C1 | 6679104 | ppvy |
| Aldehyde dehydrogenase | 13929028 | lpqy |
| Apyrase (soluble calcium-activated nucleotidase 1) | 21426787 | ppgy |
| Arachidonic acid epoxygenase (CYP2C8 ) | 13929204 | pppy |
| CYP2C29 | 9506529 | ppfy |
| CYP2D2 | 6978747 | lpiy |
| Ribophorin I | 6981486 | lpsy |
| Phosphatidylinositol 4-kinase type II | 16758554 | ppey |
| Purinergic receptor P2X4 | 13928806 | lpry |
| Similar to RIKEN cDNA 1300006M19 (BAI1-associated protein 2-like 1) | 34870394 | ppdy |
| Molecular transport ATP-binding cassette, sub-family B (MDR/TAP), member 6 | 18034785 | lpgy |
| Similar to transmembrane 9 superfamily protein member 4 | 34859018 | ppqy |
| Voltage-dependent anion channel 1 | 6755963 | ppty |
| Lysosomal acid phosphatase | 8392842 | ppgy |
| Hypothetical protein XP_236205 (SidT2) | 34863318 | lpfy |
| Similar to Chr14 ORF (TMEM55B) | 34869712 | pppy |
| Similar to FAM3C-like protein | 38454280 | ppry |
| Similar to hypothetical protein 5031407H10 (MON1B) | 34851786 | ppky |
