# Supplementary material for: A Role for the Ubiquitin Ligase Nedd4 in Membrane Sorting of LAPTM4 Proteins
Source: PLoS One. 2011 Nov 11;6(11):e27478. doi: 10.1371/journal.pone.0027478 (PMC3214061; doi:10.1371/journal.pone.0027478)
Supplement: Materials and Methods S1 — Ubiquitination Assay. Identification of PY Motif containing lysosomal proteins. (DOC) [file pone.0027478.s005.doc]

**Supporting Materials and Methods**

**Materials and Methods S1. Ubiquitination Assay**

Hek293T cells were transfected by calcium phosphate transfection. HA-LAPTM4 constructs were co-expressed with Histine-Ubiquitin (His-Ub) and/or V5-tagged Nedd4 (WT or Catalytically inactive mutant (CS)). 24 hrs post transfection cell media was exchanged and at 48 hrs the cells were lysed on ice in lysis buffer supplemented with 50 μM LLnL. Cell lysates were centrifuged at 20817 rcf (g) (30 min, 4 ^o^C). 50 μg of lysates were set aside as loading controls. 2 mg were treated with 1% SDS and boiled for 5 min to dissociate potential LATM4-interacting proteins and diluted 11 times with lysis buffer. 30 µL Nickel-Agarose beads were used to precipitate His-Ubiquitinated proteins at 4^o^C overnight. Tubes were spun at 425 rcf (g) (3 min), washed 3x with Lysis Buffer and 3x with low salt HNTG. Proteins were eluted with 30µl 1xSDS-PAGE sample buffer, resolved on 12% SDS-PAGE and transferred to nitrocellulose. The membrane was probed for the presence of ubiquitinated, HA-tagged LAPTM4.

**Materials and Methods S1. Identification of PY Motif containing lysosomal proteins**

The amino acid sequences of 215 proteins identified in a screen of rat liver tritosomes**[44]** were manually examined for the presence of PY motifs, by searching for the presence of L/PPXY sequences. Conservation of identified PY motifs in human proteins, was determined by sequence comparison using DNAassist (v3.0, University of the Free State).
